# Development and validation of a MEDLINE search filter/hedge for degenerative cervical myelopathy

**DOI:** 10.1186/s12874-018-0529-3

**Published:** 2018-07-06

**Authors:** Benjamin M. Davies, Samuel Goh, Keonwoo Yi, Isla Kuhn, Mark R. N. Kotter

**Affiliations:** 1 Department of Academic Neurosurgery, Cambrige University Hospital, University of Cambridge, Cambridge, UK; 2 University of Cambridge Medical School, University of Cambridge, Cambridge, UK; 3 University of Cambridge Medical Library, University of Cambridge, Cambridge, UK

**Keywords:** Cervical, Myelopathy, Spondylosis, Spondylotic, Stenosis, Disc herniation, Ossification posterior longitudinal ligament, Degeneration, Hedge, Search filter, MEDLINE, Systematic review

## Abstract

**Background:**

Degenerative cervical myelopathy (DCM) is a common condition with many unmet clinical needs. Pooled analysis of studies is an important tool for advancing medical understanding. This process starts with a systematic search of the literature. Identification of studies in DCM is challenged by a number of factors, including non-specific terminology and index terms. Search filters or HEDGEs, are search strings developed and validated to optimise medical literature searches. We aimed to develop a search filter for DCM for the MEDLINE database.

**Methods:**

The diagnostic test assessment framework of a “development dataset” and seperate “validation dataset” was used. The development dataset was formed by hand searching four leading spinal journals (Spine, Journal of Neurosurgery Spine, Spinal Cord and Journal of Spinal Disorders and Techniques) in 2005 and 2010. The search filter was initially developed focusing on sensitivity and subsequently refined using NOT functions to improve specificity. One validation dataset was formed from DCM narrative and systematic review articles and the second, articles published in April of 1989, 1993, 1997, 2001, 2005, 2009, 2013 and 2017 retrieved via the search MeSH term ‘Spine’. Metrics of sensitivity, specificity, precision and accuracy were used to test performance.

**Results:**

Hand searching identified 77/1094 relevant articles for 2005 and 55/1199 for 2010. We developed a search hedge with 100% sensitivity and a precision of 30 and 29% for the 2005 and 2010 development datasets respectively. For the selected time periods, EXP Spine returned 2113 publications and 30 were considered relevant. The search filter identified all 30 relevant articles, with a specificity of 94% and precision of 20%. Of the 255 references listed in the narrative index reviews, 225 were indexed in MEDLINE and 165 (73%) were relevant articles. All relevant articles were identified and accuracy ranged from 67 to 97% over the three reviews. Of the 42 articles returned from 3 recent systematic reviews, all were identified by the filter.

**Conclusions:**

We have developed a highly sensitive hedge for the research of DCM. Whilst precision is similarly low as other hedges, this search filter can be used as an adjunct for DCM search strategies.

**Electronic supplementary material:**

The online version of this article (10.1186/s12874-018-0529-3) contains supplementary material, which is available to authorized users.

## Background

Degenerative cervical myelopathy [DCM] is a new umbrella term for a common clinical phenotype: cervical spinal cord compression causing myelopathy (spinal cord damage) from degenerative changes of the surrounding spinal structures [1]. Causative degenerative pathology include disc prolapses, osteophyte formation or ligament hypertrophy. DCM is estimated to be the most common cause of spinal cord dysfunction [2] and despite surgery to alleviate compression, most patients retain lifelong disabilities. A recent study identified that quality of life amongst DCM patients was worse than patients with heart failure, COPD and Cancer [3]. Clearly therefore, there remain major unmet clinical needs in DCM.

DCM was often, formerly referred to as Cervical Spondylotic Myelopathy. However, there was inconsistency as to whether this included related conditions such as ossification of the posterior longitudinal ligament [OPLL] or ossification of the ligamentum flavum [OLF]. This has caused ambiguity in critical appraisal during research synthesis. [1] The various terms were also a mouthful for patients [4]. These factors have contributed to the proposal of DCM as a new term.

From a clinical point of view, the development of an umbrella term is logical; patients suffer the same clinical symptoms, from a presumed common spinal cord injury mechanism, undergo a similar clinical work up and are treated via surgical decompression. Therefore their pooled analysis can further understanding of diagnosis, prognosis, treatment efficacy and appropriate study design [5–8].

However, from a literature search point of view, in the absence of a recognized index term or ICD classifier, identification of relevant studies for inclusion is difficult, as the key terms are not specific to DCM. This challenges DCM research. For example:

1. Myelopathy is a medical term for disease of the spinal cord, which causes a set of common symptoms. It does not specify the aetiology or the level affected.
2. The causative pathology can occur anywhere in the spine and are common [9], more often than not incidental [10], [11, 12].
3. The type of surgical treatments for the cervical spine are common to many pathologies and not specific to DCM.
4. Cervical has anatomical relevance outside of the spine, including ‘of the cervix’, a well-researched area of women’s health.

Search filters, also known as search hedges, are validated search schemes which can be incorporated into any strategy to focus their results to a certain target. They have been developed to help clinicians efficiently and accurately filter the ever burgeoning medical literature to answer important clinical questions and advance care [13]. For example, hedges have been developed to select for specific study designs [14], specialty [15–17], themes [18] or disease [19]. Development requires testing against a manual hand search. Typically, a ‘gold standard’ database is created manually, with a proportion used for development of the hedge, which is then validated in the remainder.

Systematic reviews help prevent research wastage. In 2010 it was estimated $240 billion was spent on health research, yet as much as 85% failed to deliver meaningful clinical benefit [20]. In the report, purported reasons include duplication of existing knowledge, which could be prevented by prior systematic review.

A number of medical literature databases exist. Although Cochrane recommends the use of MEDLINE, EMBASE and CENTRAL for evaluation of interventions, MEDLINE is the most popular database and identifies the majority of included studies. It is noted, however, that the use of MEDLINE alone is often insufficient [21].

Our objective was to develop a search hedge for degenerative cervical myelopathy in the MEDLINE database. Our priority was sensitivity, with a view that from this foundation, systematic reviewers could focus their strategies.

## Method

### Study overview

**Fig. 1:**
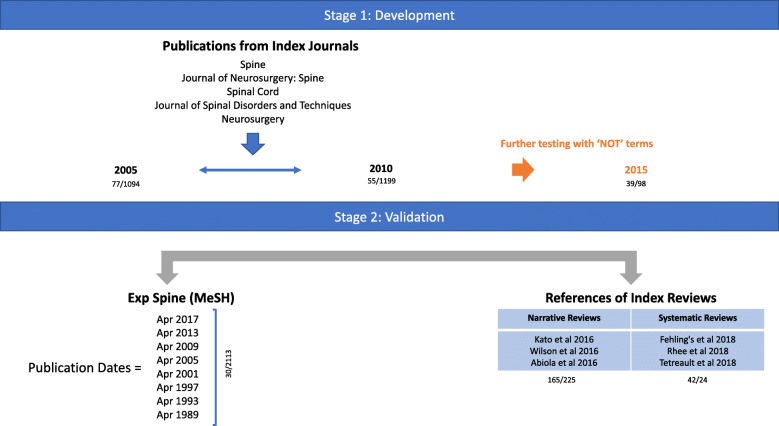
Study Overview. Three development datasets were made using the publications from lead spinal journals; *Spine, Spinal Cord, Journal of Neurosurgery: Spine, Journal of Spinal Disorder’s and Techniques and Neurosurgery.* Two separate validation data sets were created, a) using publications from April of selected years with the MeSH term ‘Spine’ and b) the references of recent DCM reviews [29–31]. The number of relevant articles is presented for each method as a fraction of the normal of articles screened

As per previous hedge development studies [15, 22], a diagnostic test assessment framework was used, whereby the hedge was developed initially using a ‘development dataset’ and subsequently validated in a ‘validation dataset’ (Fig. 1).

The objective was to develop a filter with > 98% sensitivity for studies considering any aspect of DCM in humans, for the MEDLINE database. The authors chose to focus on sensitivity, as it was intended that this filter would often form the basis of a DCM systematic review and reviewers could add additional syntax to focus their search with respect to their question.

Studies were included if they:

- Considered DCM in humans

Studies were excluded if they:

- Reported solely on animal data
- Reported on heterogeneous populations (not exclusively DCM).

Studies with heterogeneous populations are commonly identified in DCM search strategies, for as aforementioned, studies evaluating a surgical technique may use a population with mixed pathologies. The extraction of DCM specific data is therefore difficult and rarely sought. These studies were therefore excluded. However, if the study solely evaluated a surgical technique on patients with DCM, it was included.

All types of article (for example reviews, primary clinical trials or commentary), written in any language were included. As English-speaking authors, foreign language texts underwent a translated title and abstract screen only.

### Development

A development dataset was created, comprising articles published in four leading spinal journals [23] in the years 2005 and 2010; Spinal Cord, Spine, Journal of Neurosurgery Spine and Journal of Spinal Disorders and Techniques. When choosing these spinal journals, consideration was given to ensure both surgical (Spine and Journal of Neurosurgery Spine) and non-surgical (Spinal Cord and Journal of Spinal Disorders and Techniques) focused journals were selected. The DCM literature is heavily weighted towards surgery, as the only evidence-based treatment, and it was felt this balance would ensure greater generalisation of the developed search strategy given relevant material is published outside of these fields also [5, 6]. In our previous systematic reviews, 48 (44%) of the included articles were published within these four journals with the remaining 60 from 36 different journals, indicating their relevance as developmental journals to the field of DCM. Articles were hand-searched by authors (BMD, SG, KY) for inclusion using title and abstracts, and where necessary full text articles. Searches were randomly allocated to an author, such that overall, the entire database was screened at least twice by two different reviewers.

The initial search strategy was designed based on the results of our previous systematic reviews (108 relevant articles) [5, 6]. Titles and abstracts were scrutinized for relevant keywords and listed MeSH (Medical Subject Headings). The MEDLINE MeSH taxonomy was reviewed to identify appropriate grouping terms. Based on these articles, and in keeping with systematic reviews conducted by others within [8, 24–27] or related to the field [28], the filter was developed using two components: 1) ‘Pertaining to the cervical spine’ AND 2) ‘Pertaining to spinal cord compression (i.e. myelopathy)’. This was felt to be logical, as both these components have to be satisfied for a diagnosis of DCM. We sought to optimise the strategy by comparing iteration A (x number of hits) and with the subsequent iteration B (y number of hits). Where x > y, we combined A NOT B, to identify any missed papers and judge their relevance/importance. Where y > x, we combined B NOT A, to identify any missed papers and judge their relevance. Expert judgement was used to identify search terms and further optimise the search strategy.

Once a 100% sensitive search strategy had been developed, the incorporation of NOT functions was trialed to increase specificity but retain 100% sensitivity. This was undertaken using the same iterative process. In addition to the already formed developmental dataset, an additional developmental dataset was formed by screening articles identified by the 100% sensitive search strategy within the publications of the four developmental journals during 2015. If the addition of a NOT term removed a valid article, this version of the search strategy was discarded, and an alternative option trialed. In order to achieve a validated hedge with > 98% sensitive hedge (once extrapolated across less focused journals) it was believed necessary to have 100% sensitivity during development. Therefore, the loss of any relevant article was deemed unacceptable at this stage.

The filter was developed using the OVID platform for MEDLINE, in concert with a medical librarian (IK).

### Validation

Due to the relative low incidence of DCM publications, when forming our development datasets, in order to avoid hand searching extremely large datasets we had focused on leading spinal journals only. Previous hedge developments have used a single dataset, fractioned for development and validation. However to ensure that the developed hedge was generalisable to other journals, at different time points, we developed two further and distinct datasets for validation. One dataset was made up of the references from recent DCM reviews, retrievable using the OVID platform. The second dataset comprised articles published in April of 1989, 1993, 1997, 2001, 2005, 2009, 2013 and 2017 retrieved via the search EXP MeSH term ‘Spine’. The term was chosen as it is a general and broad category, but one for which DCM studies may match (Fig. 1).

### Analysis

Development and validation data sets, and the returns from filter iterations were exported from OVID, to Excel (Microsoft, California) to analyse based on their Unique Identifiers. Metrics of sensitivity, specificity, accuracy and precision were used to compare performance.

## Results

### Filter development

Hand searching of leading spinal journals identified 77 (out of 1094) relevant articles for 2005 and 55 (out of 1199) for 2010.

Optimisation of our initial search strategy, expanding on the term ‘cervical’ and ‘myelopathy’ was continued until a 100% sensitive search had been developed. An early example of this process is shown in Table 1, where the expansion of ‘cervical’ to include ‘neck’ and ‘myelopathy’ to include spinal cord injuries did not identify any more relevant articles.

**Table 1:** Example of search development and evaluation. Expanding on the terms ‘cervical’ and ‘myelopathy’ improved sensitivity. However, the use of ‘neck’ or terms relating to ‘spinal cord injury’ were of no added benefit. Results given for 2005 development database, for which 77 articles were identified using the hand search. Changes to the search strategy from one iteration to the next are shown in bold

Iteration	Search Strategy	Articles Returned	Relevant Articles	Sensitivity (%)	Precision (%)
1	(cervical and myelopathy).mp.	48	37	48%	77%
2	(**exp Cervical Vertebrae/ or exp Cervical Cord/ or** cervical.mp. **or (phrenic nucleus or accessory nucleus).mp.**) and (myelopath*.mp. or exp Spinal Cord Diseases/ or (spinal cord adj3 (diseas* or disorder*)).mp.)	56	6	56%	41%
3	(exp Cervical Vertebrae/ or exp. Cervical Cord/ or cervical.mp. or (phrenic nucleus or accessory nucleus).mp.) and (myelopath*.mp. or exp. Spinal Cord Diseases/ or (spinal cord adj3 (diseas* or disorder*)).mp. or myeloradiculopath*.mp. or (Spinal Cord adj3 Compress*).mp. or exp Spinal Cord Compression/)	7	2	58%	41%
4	(exp Cervical Vertebrae/ or exp. Cervical Cord/ or cervical.mp. or (phrenic nucleus or accessory nucleus).mp. or exp Neck/ or neck*.mp.) and (myelopath*.mp. or exp. Spinal Cord Diseases/ or (spinal cord adj3 (diseas* or disorder*)).mp. or myeloradiculopath*.mp. or (Spinal Cord adj3 Compress*).mp. or exp. Spinal Cord Compression/)	1	0	58%	40%
5	(exp Cervical Vertebrae/ or exp. Cervical Cord/ or cervical.mp. or (phrenic nucleus or accessory nucleus).mp. or exp. Neck/ or neck*.mp.) and (myelopath*.mp. or exp. Spinal Cord Diseases/ or (spinal cord adj3 (diseas* or disorder*)).mp. or myeloradiculopath*.mp. or (Spinal Cord adj3 Compress*).mp. or exp. Spinal Cord Compression/ or (spinal cord adj3 (injur* or trauma* or contusion* or lacerat*)).mp or exp Spinal Cord Injuries/)	5	0	58%	39%

The terms related to ‘cervical’ and ‘myelopathy’ had a ceiling affect, with the missing studies largely focussed on OPLL exclusively. On this basis, the ‘cervical’ component was initially expanded to include OPLL as a MeSH term and keyword, but this still missed relevant articles. As such the search strategy was expanded to include all articles with OPLL as a MeSH term, independent of the ‘Myelopathy’ search component. Another important adaptation was the inclusion JOA (Japanese Orthopaedic Association) within the ‘myelopathy’ search component, as a number of relevant articles did not mention myelopathy in their title or abstract. The JOA, in its various forms, is the most commonly used grading assessment for human function in DCM [6]. It was specifically developed for assessment of function in DCM and therefore felt to be an appropriate synonym.

With a 100% sensitive search for our development databases, we explored the use of ‘NOT’ functions to improve precision. The use of keywords was ineffective, as a number of relevant articles specified exclusion criteria in their abstract – therefore these double negatives led to inappropriate removal of relevant articles from our search results. As such, only MeSH terms could be used in the NOT search component. This reduced the number of retrieved articles by 30%, from 15, 827 to 11, 033 (searched performed 14th July 2017). The final filter for validation had a precision of 30 and 29% for the 2005 and 2010 development datasets.

### Filter validation

**Fig. 2:**
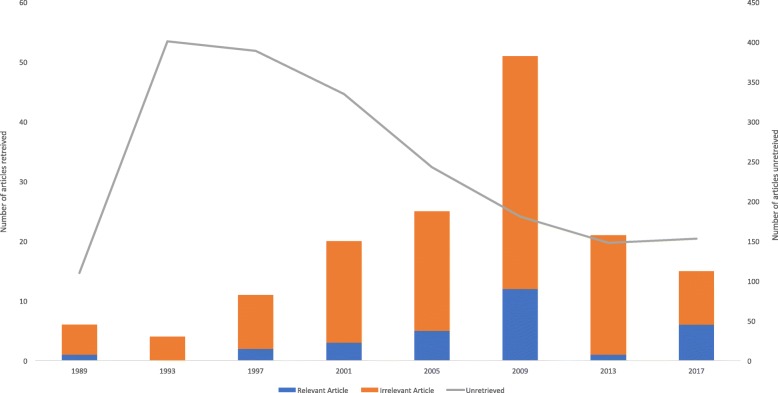
Validation in EXP Spine. The number of articles published in April, with MeSH term Spine, has decreased over time, although the proportion of relevant articles has increased. The filter had 100% sensitivity, with a precision of 5–40%

A search of MeSH Spine/ returned 23,200 articles, of which 2113 were published in April of 1989, 1993, 1997, 2001, 2005, 2009, 2013 and 2017 and 30 were considered relevant. The search filter identified all 30 relevant articles, with a specificity of 94%. Relevant articles were more likely to be published in recent years (Fig. 2). The overall precision was 20%.

We selected three recent index narrative reviews [29–31]. As a recently coined term, there were only four reviews providing an overview of DCM in MEDLINE [1, 30–32] at the time of testing. We selected the two which were not published in journals used for the HEDGE development. In addition, given the limitations identified in finding relevant OPLL articles, we also considered a recent OPLL review [29]. Of the 255 references listed, 225 were indexed in MEDLINE and 165 (73%) were relevant articles. These references spanned more than 30 years of publications, from 154 different journals (Table 2). All relevant articles were identified. Accuracy ranged from 67 to 97% over the three reviews.

**Table 2:** Validation in selected narrative review articles. All relevant references were identified with the search filter

Review Article	Number of References (Relevant Articles)	Number of Journals (Publication Years, Range)	Number of Development Journals (%)	Sensitivity (%)	Accuracy (%)
Kato et al. (2016) [33]	113 (69)	99 (2013–2016)	24 (35)	100	77
Wilson et al. (2017) [32]	84 (64)	31 (1956–2016)	33 (52)	100	97
Abiola et al. (2016) [31]	58 (32)	24 (1978–2015)	24 (75)	100	69

In addition, the included articles from the 3 most recent systematic reviews on DCM were used [33–35]. As per the objectives of the reviews, these were reviews for which the DCM search filter is an intended user. The reviews were not published in journals used as part of the development dataset. Of the 42 separate articles included (43 were included, but one study was included in two systematic reviews) all studies were identified by our search filter. 23 (53%) of included articles were published in journals used to build the development dataset.

Amongst the narrative review references, common irrelevant articles related to a surgical technique not unique to DCM (25) or OPLL in other areas of the spine (8). Surgical technique articles were appropriately not retrieved in 8 cases.

## Discussion

We have developed and validated a highly sensitive search filter for DCM (Additional file 1). Whilst the precision is relatively low, this foundation can be developed by researchers to focus their literature searches as required and is comparable to other filters [19, 36].

### How does the precision compare to other filters?

Imprecision is a recognised challenge for search filters and to be expected, when there is a relatively low proportion of relevant articles within diverse databases [37].

The use of NOT functions has been shown to improve precision. There are recognised risks of using NOT functions including a tendency to introduce errors within long search syntax and the risk of removing relevant articles [38]. Specifically, we found that the NOT functions when used with keywords removed relevant articles by creating double negatives. Specifically, some abstracts reported their exclusion criteria and therefore by also placing our matching exclusion criteria in our search syntax, the article was not returned. We do not think this has been specifically described before. Whilst this could be circumvented by using MeSH terms as opposed to keywords, this limited the terminology that could be incorporated in the NOT functions.

The precision of existing filters varies greatly, depending on the target. When evaluating filters to identify systematic reviews, Lee et al. found precision of < 5% in all tested filters [14, 39], whereas filters to identify Chronic Kidney Disease studies or content relevant for Geriatrics had precisions > 90% [16, 19, 36]. Our precision ranged from 20 to 30% depending on the database, which appears middle of the road. Efforts to further improve this using NOT functions led to the exclusion of relevant articles, which we deemed unacceptable. Although a limited comparison (considering included articles, across many databases), the precision of recent DCM reviews has been less than 8% [8, 24–27]. Given our search filter could identify the same articles, this would suggest that it would be able to increase efficiency for DCM systematic review.

### What is generating imprecision in our DCM filter?

For our search, the majority of studies that were retrieved, but irrelevant, concerned Spinal Cord Injury, surgical techniques which are shared between DCM and other conditions, such as disc replacement surgery and anterior cervical discectomy, and OPLL outside the cervical spine. Spinal cord injury and surgery for degenerative spinal disease have a high research output and bear a close relation to DCM, in terms of the contributory pathology, the disability and the treatments. As such they share many MeSH terms, and equally as often stated in DCM studies’ exclusion criteria, the use of keywords led to double negatives. However, these topics were not completely represented by our search filter, as evidenced by their mixed identification during validation, and this must be recognised by researchers wishing to use the filter. For example, if a review was to evaluate a surgical technique used in the treatment of DCM, including data from its use in other indications or wishing to extract outcomes from mixed populations, this filter would not be appropriate. If a review wanted to evaluate a technique reported just in patients with DCM, then this filter would be appropriate.

### Is imprecision a problem?

Efficiency is important for day-to-day uptake of filters, outside research fields. It is estimated that whilst a researcher may spend on average 1 h critically appraising the literature, clinically physicians may have less than 2 min to identify relevant evidence [40]. A balance between precision and sensitivity therefore needs to be struck.

Automated data mining, including the use of machine learning, for literature searching is a growing field of research [41–43]. It offers the potential to very accurately select articles of relevance and improve efficiency. At present, the areas closest to use by non-specialists, are tools to improve the efficiency of title and abstract screening in systematic reviews. These include Abstrackr [44], Revis [41] and EPPI Reviewer tools [42]. These are tools which could be used to optimise a sensitive search filter.

Our objective was to develop a search filter for DCM in MEDLINE that could form the basis of any DCM systematic review and therefore we prioritised sensitivity over all other performance metrics. Whilst this limits its usability for clinicians on a day to day basis, the intention is that no relevant studies are excluded, and that the adjuvant search strategy will improve the specificity / precision for the researchers’ needs.

### What are the limitations of this filter?

The generalisation of a search filter will be dependent on the database it was developed from. Investigators balance creating a database which is representative of the literature as a whole, with including sufficient target articles and remaining of a size which is amenable to hand searching. Various strategies have been employed; most commonly author selected journals, but also citation chasing and systematic reviews. [18]

This initial author selection process is therefore a potential limitation to generalisation. In this study, we used a combination of previous strategies, including key journals, an exploded MESH term, narrative and systematic reviews, to minimize this risk. In addition, given the search filter’s performance was maintained across many other journals and years of medical literature during validation, we therefore feel confident it is generalisable for MEDLINE.

This filter has been developed based on the presently available constructs and MeSH terms. In the absence of text mining software, frequency text analysis could not be employed to help identify key search terms. This process instead took place by visual inspection by multiple authors. Additionally if new MeSH terms arose within the field, this could alter the performance and re-validation may be needed. By incorporating both MeSH terms and free text searches we hope to ensure some longevity to this function.

## Conclusion

We have developed a highly sensitive search filter of relevance to clinical DCM research. When using this filter, it is important to consider its limitations with respect to a review’s desired objectives.

## Additional file

Additional file 1: Final, validated search filter for DCM in MEDLINE. (DOCX 15 kb)

## Abbreviations

COPD: Chronic obstructive pulmonary disease
DCM: Degenerative cervical myelopathy
JOA: Japanese Orthopaedic Association
MeSH: Medical Subject Headings
OLF: Ossification of the ligamentum flavum
OPLL: Ossification of the posterior longitudinal ligament
