# Supplementary material for: Development and validation of a MEDLINE search filter/hedge for degenerative cervical myelopathy
Source: BMC Med Res Methodol. 2018 Jul 6;18:73. doi: 10.1186/s12874-018-0529-3 (PMC6034255; doi:10.1186/s12874-018-0529-3)
Supplement: Supplementary file 1 — Final, validated search filter for DCM in MEDLINE. (DOCX 15 kb) [file 12874_2018_529_MOESM1_ESM.docx]

**Additional File 1 :** Final, validated search filter for DCM in MEDLINE

1. exp Cervical Vertebrae/ or exp Cervical Cord/ or cervical.mp. or (phrenic nucleus or accessory nucleus).mp. or (("Japanese Orthop?edic Association" adj2 score*) or (joa adj2 score*)).mp.
2. myelopath*.mp. or exp Spinal Cord Diseases/ or (spinal cord adj3 (diseas* or disorder*)).mp. or myeloradiculopath*.mp. or spondylomyelopath*.mp. or spondylomyeloradiculopath*.mp. or (Spinal Cord adj3 Compress*).mp. or exp Spinal Cord Compression/
3. 1 and 2
4. exp "Ossification of Posterior Longitudinal Ligament"/
5. 3 or 4
6. exp Atlanto-Occipital Joint/ or exp Arteriovenous Fistula/ or exp Radiotherapy/ or exp Vitamin B 12/ or exp Radiation/ or exp Radiation Injuries/ or exp Re-Irradiation/ or exp Craniospinal Irradiation/ or exp Whole-Body Irradiation/ or exp Motor Neuron Disease/ or exp Amyotrophic Lateral Sclerosis/ or exp Neoplasm Metastasis/ or exp Hemangioma/ or exp neoplasm/ or exp metastasis/ or exp Nervous System Malformations/ or exp "autoimmune diseases of the nervous system"/ or exp "congenital, hereditary, and neonatal diseases and abnormalities"/ or exp virus diseases/
7. 5 not 6
